# Phenolic Compounds and In Vitro Antibacterial and Antioxidant Activities of Three Tropic Fruits: Persimmon, Guava, and Sweetsop

**DOI:** 10.1155/2016/4287461

**Published:** 2016-08-25

**Authors:** Li Fu, WenQing Lu, XiaoMin Zhou

**Affiliations:** Liwan District Center for Disease Control and Prevention, Guangzhou 510176, China

## Abstract

In our previous study, we have found that persimmon, guava, and sweetsop owned considerably high antioxidant activity and contained high total phenolic contents as well. In order to further supply information on the antibacterial and antioxidant activity of these three tropic fruits, they were extracted by 80% methanol. We then examined the extractions about their phenolic compounds and also studied the extractions and phenolic contents about their minimum inhibitory concentration (MIC) and minimum bactericidal concentration (MBC) against twelve targeted pathogens including 8 standard strains (*Staphylococcus aureus*,* Bacillus cereus*,* Staphylococcus epidermidis*,* Monilia albican*,* Escherichia coli*,* Salmonella typhimurium*,* Shigella flexneri*, and* Pseudomonas aeruginosa*) and 4 multidrug-resistant strains (*methicillin-resistant Staphylococcus aureus*, ESBLs-producing* Escherichia coli*, carbapenems-resistant* Pseudomonas aeruginosa*, and multidrug-resistant* Acinetobacter baumannii*), which are common and comprehensive in clinic. We also employed two ways, that is, FRAP and TEAC, to evaluate their antioxidant activities, using ultraviolet and visible spectrophotometer. Our study indicated that the three tropical fruits possessed obvious antioxidant and antibacterial activity, which supported the possibility of developing the fruits into new natural resource food and functional food as well as new natural antimicrobial agent and food preservatives. Moreover, phenolic compounds detected in the fruits could be used as a potential natural antibacterial agent and antioxidant.

## 1. Introduction

The discovery of antimicrobial drugs was a monumental event in human medicine history. In recent years, however, with the widespread use of antibiotics, more and more adverse factors, such as antimicrobial resistance, have attracted the attention of researchers, pharmaceutical company, and even common people. In addition, the increasing worries about negative effects of synthetic food antioxidants and growing good wishes for pursuit of better healthy life prompt scientists to research to replace synthetic medicines and antioxidants.

It is pleasing for scientists to be equipped with a new option from studying of natural plant in this field. Actually, in many countries, such as China, India, and Japan, plants have been used to enhance health and even as medicinal cure for thousands of years.

In our previous study, we tested 62 fresh fruits that were sold in Guangzhou market and found that persimmon, guava, and sweetsop had very high antioxidant activity and high total phenolic contents [1]. Based on the study, we selected persimmon, guava, and sweetsop as the objects, analyzed their phenolic compounds, and studied their antibacterial activity against twelve common pathogenic bacteria. At the same time, we researched the antioxidant activities of fresh and dried fruits and the phenolic compounds.

Persimmon is a native fruit of China. The persimmon tested grows in Conghua, Guangzhou. It is cylindrical in shape and looks like chicken heart. It was reported that persimmon could be used for treatment of diarrhea, dry coughs, and hypertension [2]. Recently, persimmon leaf-based products were used in the foot socks and soap of athlete [3].

Guava is a tropic and subtropic fruit. The most prevailing guava in Guangzhou has green peel and white pulp, whose shape is sphere and oval. It was reported that guava could benefit diabetes, caries, wounds, diarrhea, inflammation, and hypertension. The extracts from guava showed antispasmodic, anti-inflammatory, anticancer, antioxidant, and hypoglycemic effect [4, 5].

Sweetsop was native to tropic America and introduced to China about 400 years ago. It has much medicinal use such as against oxidative and hepatic damage, tumors, diabetes, epilepsy, cardiac problems, constipation, and ulcers [6]. Meanwhile, sweetsop had beneficial effects on the formation of glycosaminoglycans and collagen during wound healing [7].

Fruits and vegetables had many kinds of health-promoting compounds such as high concentrations of phenolic compounds, vitamins, fiber, and minerals, among which phenolic compounds might protect people from some chronic diseases, although they were not essential for life sustaining [8]. Phenolic compounds have been demonstrated to be an antioxidant better than vitamins E and C because they have the ability to chelate metal ions and be electron donors [9]. The study of Sun found that diseases such as mutagenesis and carcinogenesis might be inhibited as long as 1 g of phenolic compounds is taken every day from fruits and vegetable which are rich in antioxidants [10]. Taking all these pieces of information into consideration, we designed this study aiming at understanding the phenolic compounds that consisted of persimmon, guava, and sweetsop growing in south of China and evaluating the antibacterial and antioxidant activity of the fruit and phenolic compounds.

## 2. Experimental Section

### 2.1. Materials and Sample Preparation

Phenolic compounds standards, including gallic acid, (+)-catechin, (−)-epicatechin, chlorogenic acid, caffeic acid, ferulic acid, quercetin, luteolin, kaempferol, TPTZ (2,4,6-tri(2-pyridyl)-s-triazine/tripyridyltriazine), Trolox (6-hydroxy-2,5,7,8-tetramethylchroman-2-carboxylic acid), and ABTS (2,2-azino-di-(3-ethylbenzothiazoline-sulfonic acid)), were purchased from Sigma-Aldrich. All the chemicals were of analytical grade or better. Mueller-Hinton Broth and nutrient agar were purchased from Guangdong Huankai Microbial SCI. & TECH. Co. Ltd. (China).

Fresh and mature fruits were selected and bought in Guangzhou market. The samples for antioxidant study could be fresh or dry, but those for antibacterial study and HPLC analysis should only be dried. The fruits were washed with deionized water before being peeled and cut into slices. Then they were put into the oven to be dried at 60°C for 48 hours and ground before being passed through a 40-mesh sieve to produce dry samples. Around 2.0 g dry samples or slurry of edible portion from fresh samples was ultrasonic extracted with 20 mL methanol-water (80 : 20, v/v) at room temperature for 30 min, then refluxed, and collected into 20 mL. The collection was centrifuged at 12000/min, 4°C for 10 min. The supernatant from dried samples was used for the evaluation of HPLC analysis, and those from dried and fresh samples were used for antioxidant capacity study. In order to get samples with high concentration for antibacterial study, about 50.0 g dried samples were ultrasonic extracted with methanol-water (80 : 20, v/v) at room temperature for 60 min. The extraction was filtered and the collected fluid was concentrated to 5.0 g/mL for sweetsop and 2.5 g/mL for persimmon and guava. All the samples were stored at 4°C until being used for experiment. To the phenolic compounds detected in the fruit, 5 mg/mL solution of each standard substance was used for antibacterial study.

### 2.2. HPLC Protocol

Separation was carried out using Diamosil (R) C18 column (250*∗*4.6 mm 5 *μ*m) at 35°C. The gradient elution solution consisted of solvents A (methanol) and B (pH 3 formic acid-water) as the following program: 5 min, 80% B; 10 min, 65% B; 20 min, 60% B; 25 min, 30% B; 30 min, 25% B; 40 min, 90% B, and it finished at 48 min. Stock solution of phenolic compounds standards (1000 *µ*g/mL) was prepared in 80% methanol (some DMSO added to help dissolve quercetin, luteolin, and kaempferol), while calibration standards (2.5–100 *µ*g/mL) were prepared from the stock solution by serial dilutions. All of the samples were filtered through 0.45 *µ*m nylon syringe filters before analysis.

### 2.3. Microorganisms and Inocula Preparation

Eight standard strains included four Gram-positive bacteria, that is,* Staphylococcus aureus* CMCC(B)26003,* Bacillus cereus* CMCC(B)63301,* Staphylococcus epidermidis* CMCC(B)26069, and* Monilia albican* CMCC(F)98001, and four Gram-negative bacteria, that is,* Escherichia coli* ATCC25922,* Salmonella typhimurium* CMCC(B)50115,* Shigella flexneri* CMCC(B)51572, and* Pseudomonas aeruginosa* ATCC27853. They were obtained from Guangdong Huankai Microbial SCI. & TECH. Co. Ltd. Four multidrug-resistant strains (i.e., MRSA, ESBLs-producing* Escherichia coli*, carbapenems-resistant* Pseudomonas aeruginosa*, and multidrug-resistant* Acinetobacter baumannii*) were provided by Laboratory Department of Nanfang Hospital. The bacterial strains were cultured at 37°C in Mueller-Hinton Broth. The bacterial suspension was adjusted to 0.5 McFarland standards by turbidity and then diluted by 1 : 10 for two times.

### 2.4. Determination of Minimum Inhibitory Concentration (MIC)

Twofold microdilution broth method [11] was used to determine the MIC in this study. All the samples were diluted by deionized water except quercetin, luteolin, and kaempferol, which were diluted by 5% dimethyl sulfoxide (DMSO) because of their poor solubility in water. 70 *µ*L of each sample solution was added to the sterile 96 wells that contained 70 *µ*L Mueller-Hinton Broth and 70 *µ*L bacterial suspension. After the samples being vibrated and mixed, the absorbance was checked. Then the samples were cultivated in 37°C incubator for 24 hours, and their absorbance was checked again. The microdilution trays were inspected from the minimum concentration with naked eyes to detect the growth inhibition of the bacteria.

### 2.5. Determination of Minimum Bactericidal Concentration (MBC)

200 *µ*L sample solutions were transferred from each well that was found to inhibit visible microorganism growth to Mueller-Hinton agar plates and then incubated at 37°C for 24 hours. The lowest concentration of samples without viable bacteria being identified was defined as MBC [12].

### 2.6. Ferric-Reducing Antioxidant Power (FRAP) Assay

The FRAP assay was carried out according to the literature with slight modifications [13]. Briefly, the FRAP reagent was prepared from sodium acetate buffer (300 mM, pH 3.6), 10 mM TPTZ solution (40 mM HCl as solvent), and 20 mM iron(III) chloride solution at volume ratio of 10 : 1 : 1, respectively. The FRAP reagent was prepared freshly daily and warmed to 37°C in a water bath before use. 100 *µ*L of the diluted sample was added to FRAP reagent (3 mL). After 4 min incubation, the absorbance of the reaction mixture was measured at 593 nm using ultraviolet-visible spectrophotometer. The standard curve was constructed using FeSO_4_ solution, and the results were expressed as *µ*mol Fe^2+^/g wet or dry weight of sample.

### 2.7. Trolox Equivalent Antioxidant Capacity (TEAC) Assay

The TEAC assay was determined according to the method with minor modifications [14]. Briefly, the ABTS^∙+^ stock solution was prepared from 7 mM ABTS and 2.45 mM potassium persulfate at volume ratio of 1 : 1, and then incubated in dark for 16 h at room temperature, being used within 2 days. The ABTS^∙+^ working solution was prepared by diluting the stock solution with ethanol to an absorbance of 0.70 ± 0.05 at 734 nm. All samples were diluted approximately to supply 20–80% inhibition of the blank absorbance. 100 *µ*L of the diluted sample was mixed with ABTS^∙+^ working solution (3.8 mL) and, after 6 min of incubation at room temperature, the absorbance of the reaction mixture was measured at 734 nm. Then the percent of absorbance inhibition at 734 nm was calculated. Trolox was used as a reference standard, and the results were expressed as *µ*mol Trolox/g wet or dry weight of sample.

### 2.8. Statistical Analysis

All the experiments were performed in triplicate, and the results were expressed as mean ± SD (standard deviation). Statistical analysis was performed using Excel 2007 and SPSS 13.0.

## 3. Results and Discussion

### 3.1. Phenolic Compounds in the Three Fruits

There were 8 phenolic compounds detected in this study, only caffeic acid excluded. The contents of different phenolic compounds in persimmon, guava, and sweetsop were showed in Table 1 and varied with the difference of 13-, 10-, and 6-fold, respectively. For persimmon, five phenolic compounds were detected and the one with the highest content was gallic acid (377.11 ± 18.47 mg/Kg), followed by (+)-catechin (125.29 ± 9.61 mg/Kg) and quercetin (102.65 ± 4.96 mg/Kg), while the one with the lowest was kaempferol (29.16 ± 1.16 mg/Kg). The content of gallic acid in persimmon was about 4 times as high as that in guava. There were six phenolic compounds found in guava and sweetsop. For guava, (+)-catechin showed the highest content (391.93 ± 15.08 mg/Kg) and kaempferol the lowest (38.06 ± 2.00 mg/Kg). For sweetsop, the content of gallic acid was found to be the highest (256.52 ± 14.33 mg/Kg) while chlorogenic acid was the lowest (43.56 ± 3.72 mg/Kg).

Previous study about persimmon showed that gallic, protocatechuic, caffeic, p-coumaric, and ferulic acids and myricetin were found, among which gallic acid was the most abundant phenolic compound (287.5 ± 5.31 *μ*g/g) [15]. In comparison, our results were much higher and the content of gallic acid was about 1.3-fold of that in their study. One report found that there were gallic acid, methylgallate, myricetin-3-O-beta-glucuronide, myricetin-3-O-alpha-rhamnoside, myricetin and quercetin, ellagic acid, and kaempferol in persimmon [16]. Also, myricetin, quercetin, isorhamnetin, quercitrin, 1-O-trans-cinnamoyl-*β*-D-glucopyranose, and ellagic acid were found in guava [17]. To sweetsop, protocatechuic acid, eriodictyol, quercetin, syringic acid, and stearic acid were detected [18]. All these results indicated that natural fruits contained various phenolic compounds, which were presumed to be responsible for antibacterial and antioxidant effect.

### 3.2. Antibacterial Activity

For the 8 standard strains, the three fruits showed different antimicrobial activities. As seen from Table 2, MIC of persimmon, guava, and sweetsop against Gram-positive (G+) was between 312.5 and 1250 mg/mL, 78.125 and 1250 mg/mL, and 312.5 and 625 mg/mL, respectively, while those against Gram-negative (G−) was between 625 and 1250 mg/mL, 312.5 and 2500 mg/mL, and 2500 mg/mL, separately. MBC of persimmon against G+ and G− was between 312.5 and 2500 mg/mL and 625 and 1250 mg/mL separately, and that of guava was 156.25 and 2500 mg/mL and 312.5 and 1250 mg/mL accordingly. MBC of sweetsop extraction against G+ bacteria* M. albican* and G− bacteria* S. typhimurium* was greater than the original concentration (5000 mg/mL). To the other three G+ bacteria and three G− bacteria, MBC of sweetsop was between 1250 and 2500 mg/mL and 2500 and 5000 mg/mL, respectively.

However, it is more interesting to get the data related to the 4 multidrug-resistant strains (Table 3). For persimmon, we did not find any antimicrobial activities against the four multidrug-resistant strains. Meanwhile, sweetsop also showed the similar features except that its MIC and MBC against MRSA was 5000 mg/mL. But there were exciting finds in the study of guava, whose MIC and MBC against the four multidrug-resistant strains were between 312.5 and 625 mg/mL and 312.5 and 1250 mg/mL, respectively. When compared with the results of standard strains* S. aureus*,* E. coli*, and* P. aeruginosa*, guava showed stronger or at least the same antibacterial activity against multidrug-resistant strains.

Other studies found that persimmon leaves extract showed antibacterial activity and inhibition against* E. coli* (8.7*∗*10^5^ CFU/mL) and* S. aureus* (2.2*∗*10^5^ CFU/mL), as well as* Campylobacter sputorum*,* Streptococcus mutans*, and* Bacteroides thetaiotaomicron* [3]. Guava extract was found in our study to be able to inhibit the growth of* S. aureus*,* M. albican*,* E. coli*, and* P. aeruginosa*, which was in well-agreement with results reported in the literature [19]. However,* E. coli* and* P. aeruginosa* were sensitive to all the three fruits in our study, contradicting results obtained by Qabaha [20]. Previous study showed that guava possessed the highest inhibition for* L. monocytogenes*,* S. aureus*,* S. aureus*, and* V. parahaemolyticus* [21]. Also, sweetsop extract was found to be capable of inhibiting* E. coli*,* M. albican*,* S. typhimurium*,* Klebsiella pneumoniae*, and* Proteus vulgaris* [22].

As far as the ration of MBC to MIC was concerned, bactericidal effect was defined as MBC/MIC ratio ≤4, while bacteriostatic effect was determined as MBC/MIC >4 [23]. In the study of 8 standard strains, sweetsop had bacteriostatic activity against* S. epidermidis* and bactericidal activity against* S. aureus*,* B. cereu*,* E. coli*,* S. flexneri*, and* P. aeruginosa*, while persimmon and guava exhibited bactericidal activity against all the eight pathogens. When multidrug-resistant strains were studied, sweetsop only had bactericidal activity against MRSA, while guava showed bactericidal activity against all the four strains. It was quite exciting, because the bacteria, such as* S. aureus*,* E. coli*,* S. typhimurium*, and* P. aeruginosa*, were among the most common clinical pathogens isolated from patients and could develop into multidrug-resistant strains in various circumstances [24], not to mention the clinical common multidrug-resistant strains. Therefore, our results indicated the considerable potentials of the three fruits in health enhancing and folk medicine.

We also studied the antibacterial activity of phenolic compounds detected in the three fruits. In Table 2, for the standard strains, MIC of subjects was all between 0.625 and 5 mg/mL and varied with range of more than one dilution. MBC of (+)-catechin towards* M. albican*, (−)-epicatechin towards* B. cereus *and* P. aeruginosa*, chlorogenic acid towards* S. epidermidis* and* M. albican*, and ferulic acid and luteolin towards* E. coli* was all higher than the highest test concentration (5 mg/mL). In Table 3, for the four multidrug-resistant strains, MIC and MBC of phenolic compounds were between 1.25 and 5 mg/mL and 2.5 and 5 mg/mL, respectively. Compared to standard strains, phenolic compounds against multidrug-resistant strains showed the same or slightly lower antibacterial activity, which was consistent with the clinical manifestations in much extent.

Another study suggested that antibacterial activity was in a positive correlation with the amount of phenolic compounds in the plant [25]. It was reported that gallic acid could restrain the growth of many bacteria, including methicillin-sensitive* S. aureus*, MRSA,* E. coli*,* P. aeruginosa*, and* Salmonella typhi* [26, 27]. Gallic acid and catechin have been shown to own the antibacterial activity against* Helicobacter pylori* [28]. MIC of ferulic acid against* Xylella fastidiosa* strains stayed between 800 and 2000 *µ*M, and MIC of gallic acid, quercetin, and catechin against* Xylella fastidiosa* strains lay between 200 and 400 *µ*M [29]. Luteolin, however, was found to have broad spectrum antibacterial effect against* S. aureus*,* B. subtilis*,* E. coli*, and* P. aeruginosa* [30]. Fattouch reported that kaempferol presented antibacterial activity only above concentration of 10 mg·mL^−1^, which was different from our results, and there were many factors causing the disagreement, such as different methods employed, as well as microbial strains, extraction processes, and concentrations of extracts and microbes [31].

In our study, all the phenolic compounds possessed bactericidal activity against the test strains, when MBC was no less than 5 mg/mL. The antibacterial activity of these phenolic contents confirmed by our study should involve many mechanisms, including alteration of the physicochemical properties of the plasma membrane, pore formation, inhibition of DNA gyrase and nucleic acid synthesis, and toxicity through the generation of hydrogen peroxide [4, 32]. As a result, the bactericidal effect of the three fruits against the selected strains in our study might partially owe to these phenolic compounds. Antimicrobial properties make phenolic compounds possible to be developed into the treatment of fungal and microbial infections in the future, or, at least, our study supplied considerable potentials to exploit natural agents against bacteria from these fruits.

### 3.3. Antioxidant Activity

FRAP assay is a widely used method for evaluating antioxidant capacity. However, most known natural antioxidants are not single-functional, and so it is required to introduce different antioxidant activity assessments, which can consider various mechanisms of antioxidant action [33]. Therefore, TEAC assay could be introduced as the method to evaluate the free radical scavenging capacity.

The regression equation of FeSO_4_ standard curve in FRAP assay was *y* = 0.00072*x* + 0.05170, *R* ^2^ = 0.99999, and that of ABTS standard curve in TEAC assay was *y* = 0.00093*x* − 0.00367, *R* ^2^ = 0.99993.

As seen from the data in Table 4, dried sample had higher antioxidant activity than fresh one. The antioxidant values of dried fruit were about 1.8 to 4.0 times fresh one. For TEAC, no matter fresh or dried sample, the values were in the order sweetsop > guava > persimmon, just the same as the sequence of FRAP for fresh fruits. When dried fruits were checked by FRAP, however, the values were listed as guava > sweetsop > persimmon.

It should be pointed out that the results from FRAP for fresh samples this time (12.59 ± 0.55, 26.07 ± 0.32, 34.37 ± 0.49 *μ*molFe^2+^/g) were consistent with our previous study (16.97 ± 0.26, 23.80 ± 1.44, 22.04 ± 1.05 *μ*molFe^2+^/g) basically and that those of TEAC (5.10 ± 0.08, 9.13 ± 0.42, 21.30 ± 0.92 *μ*mol Trolox/g) were also found to fit with the same previous study (9.38 ± 0.25, 15.18 ± 0.81, 23.60 ± 0.06 *μ*mol Trolox/g) approximately, which suggested that different extraction did not lead to obvious changes in terms of FRAP and TEAC [1]. It was also found that there was some difference between the values obtained from our current study and those reported in the literatures [9, 34–36].

FRAP values and TEAC values of phenolic compounds detected in the study were shown in Table 5. In general, these phenolic compounds had very high antioxidant capacities. FRAP values varied with the difference of 3-fold and gallic acid had the highest value (35.45 ± 1.46 mmolFe^2+^/g), while chlorogenic acid showed the lowest (11.33 ± 0.22 mmolFe^2+^/g). Meanwhile, chlorogenic acid was the only one whose FRAP value was slightly lower than the reference compound, ascorbic acid (12.66 ± 0.13 mmolFe^2+^/g). TEAC values varied with the difference of almost 6-fold. Similar to the result of FRAP, gallic acid had the highest TEAC value (23.56 ± 1.17 mmol Trolox/g) and chlorogenic acid the lowest (4.10 ± 0.04 mmol Trolox/g). Equally, TEAC value of chlorogenic acid was also lower than ascorbic acid (5.42 ± 0.05 mmolFe^2+^/g), while that of luteolin and kaempferol was almost similar to the reference compound.

Phenolics owned perfect chemistry for antioxidant activity because they had high reactivity to donate hydrogen or electron and were capable of chelating metal ions [37]. Our previous study found the content of phenolic was in a positive correlation with its antioxidant activity [1]. The higher the phenolic content is, the higher the antioxidant activity is.

In addition, a highly positive correlation (*R* ^2^ = 0.801) in the fruits between FRAP value and TEAC value was founded, which indicated that the fruits capable of reducing oxidants might be in accord with scavenging free radicals. But when the phenolic compounds were studied, the correlation of FRAP value and TEAC value was relatively lower (*R* ^2^ = 0.632), suggesting that, except for phenolic compounds, other bioactive ingredients, such as polysaccharides, phytochelatins, and peptides, might also display reducing oxidants power as antioxidant mechanisms. Also, we must point out that both methods we used in this study were ET-based assays which measured antioxidant's reducing capacity of the sample. ET-based assay was a significant method and could supply useful data, especially thinking that some water soluble oxidant compounds could be easily reduced to harmless substances, although it could not directly test its radical scavenging capacity. Another extremely important method was HAT-based assay which quantified hydrogen atom donating capacity and was more relevant to radical chain-breaking antioxidant capacity [38]. However, limited by apparatus, we did not study antioxidant capacity by HAT method.

## 4. Conclusions

The three kinds of tropical fruits, including persimmon, guava, and sweetsop, possessed antioxidant and antibacterial activity that supported the possibility of developing the fruits into new natural resource food and functional food as well as new natural antimicrobial agent and food preservatives. Moreover, phenolic compounds detected in the fruits could be used as a potential natural antibacterial agent and antioxidant. Further studies should be carried out to evaluate in vivo activities so that potential clinical drug and health products could be developed.

## Figures and Tables

**Table 1:** Content of phenolic compounds in the three fruits (mg/Kg).

Number	Phenolic compounds	Persimmon	Guava	Sweetsop
1	Gallic acid	377.11 ± 18.47	99.15 ± 1.62	256.52 ± 14.33
2	(+)-Catechin	125.29 ± 9.61	391.93 ± 15.08	144.06 ± 7.90
3	(−)-Epicatechin		58.43 ± 4.70	
4	Chlorogenic acid			43.56 ± 3.72
5	Caffeic acid			
6	Ferulic acid			186.05 ± 17.02
7	Quercetin	102.65 ± 4.96	122.23 ± 10.14	73.64 ± 2.43
8	Luteolin	48.97 ± 0.86	51.39 ± 3.44	73.12 ± 3.36
9	Kaempferol	29.16 ± 1.16	38.06 ± 2.00	

**Table 2:** MIC, MBC, and MBC/MIC results of the fruits and phenolic compounds to 8 standard strains.

Antibacterial properties	Phenolic compounds	Gram-positive bacteria				Gram-negative bacteria			
		*S. aureus*	*B. cereus*	*S. epidermidis*	*M. albican*	*E. coli*	*S. typhimurium*	*S. flexneri*	*P. aeruginosa*
MIC (mg/mL)	Persimmon	312.50	625.00	625.00	1250.00	1250.00	1250.00	1250.00	625.00
	Guava	1250.00	312.50	78.13	1250.00	625.00	625.00	625.00	312.50
	Sweetsop	1250.00	1250.00	312.50	2500.00	2500.00	2500.00	2500.00	2500.00
	Gallic acid	0.63	2.50	0.63	5.00	2.50	2.50	2.50	2.50
	(+)-Catechin	1.25	1.25	1.25	2.50	5.00	2.50	2.50	2.50
	(−)-Epicatechin	2.50	2.50	2.50	2.50	2.50	2.50	2.50	1.25
	Chlorogenic acid	5.00	2.50	0.63	5.00	5.00	2.50	2.50	2.50
	Ferulic acid	2.50	2.50	2.50	2.50	2.50	2.50	2.50	2.50
	Quercetin	2.50	2.50	1.25	2.50	2.50	2.50	2.50	2.50
	Luteolin	0.63	5.00	0.63	5.00	2.50	2.50	2.50	5.00
	Kaempferol	2.50	2.50	1.25	2.50	2.50	2.50	1.25	1.25

MBC (mg/mL)	Persimmon	312.50	625.00	625.00	2500.00	1250.00	1250.00	1250.00	625.00
	Guava	2500.00	312.50	156.25	2500.00	1250.00	625.00	625.00	312.50
	Sweetsop	2500.00	1250.00	2500.00	>5000.00	5000.00	>5000.00	5000.00	2500.00
	Gallic acid	0.63	2.50	0.63	5.00	5.00	5.00	5.00	5.00
	(+)-Catechin	1.25	1.25	1.25	>5.00	5.00	2.50	2.50	2.50
	(−)-Epicatechin	2.50	>5.00	5.00	5.00	2.50	2.50	2.50	>5.00
	Chlorogenic acid	5.00	2.50	>5.00	>5	5.00	5.00	5.00	2.50
	Ferulic acid	5.00	2.50	5.00	5.00	>5.00	5.00	5.00	5.00
	Quercetin	5.00	2.50	2.50	5.00	5.00	2.50	5.00	5.00
	Luteolin	2.50	5.00	1.25	5.00	>5.00	5.00	5.00	5.00
	Kaempferol	5.00	2.50	1.25	5.00	5.00	5.00	5.00	5.00

MBC/MIC	Persimmon	1	1	1	2	1	1	1	1
	Guava	2	1	2	2	2	1	1	1
	Sweetsop	2	1	8	/	2	/	2	1
	Gallic acid	1	1	1	1	2	2	2	2
	(+)-Catechin	1	1	1	/	1	1	1	1
	(−)-Epicatechin	1	/	2	2	1	1	1	/
	Chlorogenic acid	1	1	/	/	1	2	2	1
	Ferulic acid	2	1	2	2	/	2	2	2
	Quercetin	2	1	2	2	2	1	2	2
	Luteolin	4	1	2	1	/	2	2	1
	Kaempferol	2	1	1	2	2	2	4	4

Note: “/” means the MBC/MIC result cannot be provided because MBC was greater than the original concentration.

**Table 3:** MIC, MBC, and MBC/MIC results of the fruits and phenolic compounds to 4 multidrug-resistant bacteria (mg/mL).

Samples	MRAS			ESBL *E. coli*			Carbapenems-resistant* P. aeruginosa*			Multidrug-resistant *A. baumannii*		
	MIC	MBC	MBC/MIC	MIC	MBC	MBC/MIC	MIC	MBC	MBC/MIC	MIC	MBC	MBC/MIC
Persimmon	/	/	/	/	/	/	/	/	/	/	/	/
Guava	312.50	312.50	1	625.00	1250.00	2	312.50	312.50	1	312.50	312.50	1
Sweetsop	5000.00	5000.00	1	/	/	/	/	/	/	/	/	/
Gallic acid	2.50	2.50	1	2.50	5.00	2	2.50	2.50	1	2.50	2.50	1
(+)-Catechin	1.25	2.50	2	2.50	2.50	1	2.50	5.00	2	2.50	2.50	1
(−)-Epicatechin	2.50	2.50	1	2.50	5.00	2	2.50	5.00	2	2.50	2.50	1
Chlorogenic acid	2.50	5.00	2	2.50	2.50	1	2.50	2.50	1	2.50	2.50	1
Ferulic acid	2.50	2.50	1	2.50	2.50	1	2.50	2.50	1	2.50	2.50	1
Quercetin	2.50	2.50	1	5.00	5.00	1	1.25	2.50	2	1.25	2.50	2
Luteolin	2.50	2.50	1	2.50	2.50	1	1.25	2.50	2	2.50	2.50	1
Kaempferol	2.50	5.00	2	2.50	2.50	1	5.00	5.00	1	2.50	2.50	1

Note: “/” means the MIC, MBC, and MBC/MIC result cannot be provided.

**Table 4:** FRAP and TEAC results of the persimmon, guava, and sweetsop.

Fruit		FRAP (*μ*mol Fe^2+^/g)	TEAC (*μ*mol Trolox/g)
Fresh	Persimmon	12.59 ± 0.55	5.10 ± 0.08
	Guava	26.07 ± 0.32	9.13 ± 0.42
	Sweetsop	34.37 ± 0.49	21.30 ± 0.92

Dried	Persimmon	51.50 ± 1.51	20.14 ± 0.97
	Guava	95.25 ± 3.99	31.91 ± 2.54
	Sweetsop	71.54 ± 2.69	37.32 ± 0.75

**Table 5:** FRAP and TEAC results of phenolic compounds.

Phenolic compounds	FRAP (mmol Fe^2+^/g)	TEAC (mmol Trolox/g)
Gallic acid	35.45 ± 1.46	23.56 ± 1.17
(+)-Catechin	13.41 ± 0.26	12.64 ± 0.18
(−)-Epicatechin	13.91 ± 0.12	13.00 ± 0.06
Chlorogenic acid	11.33 ± 0.22	4.10 ± 0.04
Ferulic acid	15.56 ± 0.36	13.19 ± 0.07
Quercetin	25.96 ± 0.06	13.32 ± 0.57
Luteolin	14.87 ± 0.60	5.22 ± 0.60
Kaempferol	15.90 ± 0.14	5.32 ± 0.02

Note: the result of FRAP and TEAC about ascorbic acid tested in this study is 12.66 ± 0.13 mmol Fe^2+^/g and 5.42 ± 0.05 mmol Trolox/g, respectively.
